# Application of a Neural Network Classifier to Radiofrequency-Based Osteopenia/Osteoporosis Screening

**DOI:** 10.1109/JTEHM.2021.3108575

**Published:** 2021-08-30

**Authors:** Johnathan W. Adams, Ziming Zhang, Gregory M. Noetscher, Ara Nazarian, Sergey N. Makarov

**Affiliations:** 1 Department of Electrical and Computer EngineeringWorcester Polytechnic Institute 8718 Worcester MA 01609 USA; 2 Neva Electromagnetics LLC Yarmouth Port MA 02675 USA; 3 Musculoskeletal Translational Innovation InitiativeCarl J. Shapiro Department of Orthopaedic SurgeryBeth Israel Deaconess Medical Center, Harvard Medical School Boston MA 02215 USA; 4 Department of Orthopedic SurgeryYerevan State Medical University 0025 Yerevan Armenia; 5 Athinoula A. Martinos Center for Biomedical Imaging, Massachusetts General HospitalHarvard Medical School 1811 Boston MA 02114 USA

**Keywords:** Artificial intelligence, neural networks, osteopenia, osteoporosis, radiofrequency measurements, signal processing

## Abstract

Objective: There is an unmet need for quick, physically small, and cost-effective office-based techniques that can measure bone properties without the use of ionizing radiation. Methods: The present study reports the application of a neural network classifier to the processing of previously collected data on very-low-power radiofrequency propagation through the wrist to detect osteoporotic/osteopenic conditions. Our approach categorizes the data obtained for two dichotomic groups. Group 1 included 27 osteoporotic/osteopenic subjects with low Bone Mineral Density (BMD), characterized by a Dual X-Ray Absorptiometry (DXA) T-score below – 1, measured within one year. Group 2 included 40 healthy and mostly young subjects without major clinical risk factors such as a (family) history of bone fracture. We process the complex radiofrequency spectrum from 30 kHz to 2 GHz. Instead of averaging data for both wrists, we process them independently along with the wrist circumference and then combine the results, which greatly increases the sensitivity. Measurements along with data processing require less than 1 min. Results: For the two dichotomic groups identified above, the neural network classifier of the radiofrequency spectrum reports a sensitivity of 83% and a specificity of 94%. Significance: These results are obtained without including any additional clinical risk factors. They justify that the radio transmission data are usable on their own as a predictor of bone density. This approach has the potential for screening patients at risk for fragility fractures in the office, given the ease of implementation, small device size, and low costs associated with both the technique and the equipment.

## I. Introduction

Approximately 50% of women and 20% of men over the age of 50 will suffer from a fragility fracture in their remaining lifetime [1]. Hip fracture is one of the most serious and debilitating outcomes of osteoporosis [2], [3], with a 14–36% mortality rate during the first year post-fracture [4]. Hip fracture incidence rates are known to increase exponentially with age in both women and men [5], and with the rising life expectancy throughout the globe, osteoporosis is expected to increase to 14 million cases with over 47 million cases of low bone mass density by 2020. Thus, the number of fractures is predicted to double or triple by 2040 [6].

The World Health Organization (WHO) has defined individuals at risk for these fractures based on their areal Bone Mineral Density (aBMD, g/cm2) relative to that of a normal young adult, as measured by Dual-energy X-ray Absorptiometry (DXA). Some shortcomings of DXA include: exposing patients to small ionizing radiation doses of up to 0.86 mrem [7]; the surrounding soft tissues can introduce relevant measurement errors [8], [9]; bone mineral density (BMD) measurements are affected by variations in bone size [10], [11]; and cortical and trabecular bone cannot be separated [12]. Additionally, fracture predictions based on aBMD are neither sensitive nor specific [13]–[17]. A DXA exam requires a visit to a hospital and the use of a room-scale, static machine with a skilled operator [18].

Quantitative ultrasound has been used as a low-cost, non-ionizing technique to screen patients for osteoporosis, employing a dedicated scanner to acquire data predominantly at the calcaneus. A commercial ultrasound device Bindex^®^ uses the pulse-echo technique to measure the thickness of the frontal cortical shell of the tibia bone [19]–[22]. These measurements have been found to correlate well with DXA measurements [19].

Microwave or radiofrequency imaging of (heel) bone was first introduced by Dr. Keith Paulsen and his research group at Dartmouth College approximately ten years ago as an alternative non-ionizing diagnostic method to assess bone health [2], [23]–[26]. Due to the well-known complexity and poor spatial resolution of the standard microwave imaging setup [27], [28] used in these studies, no clinically applicable results have been generated to date. However, the underlying physical idea of this method is simple and powerful. In osteoporosis, bone mass decreases and pore size increases. The lost bone mass is replaced by a mixture of yellow bone marrow. Such substantial changes in physical properties must alter electromagnetic tissue properties [29], [30] and must generate a significantly different radio-frequency (RF) channel through the bone. It may therefore be sufficient to track an integral measure of radio wave propagation along the path through the bone instead of restoring the complete permittivity map, as attempted previously [2], [23]–[26].

To do so, we have selected the wrist, a body compartment where bone constitutes a significant fraction of the total tissue volume and is easily accessible. We have designed on-body transmitting/receiving dual antiphase patch antennas with controlled pressure on this anatomic site [31]. We have further measured radio wave propagation through this compartment and compared our results with osteoporotic and osteopenic (low bone density) conditions established via DXA and through a history of bone fracture [32].

The perceptron-style neural network was first published in 1957 by Rosenblatt [33]. Since then, neural networks have proven beneficial in the analysis of complex datasets involving frequency spectra [34]–[36]. Additionally, the diagnosis of osteoporosis using neural networks is not unprecedented [37]–[40]. Prior works using neural networks to predict osteoporosis diagnosis focus on the aggregation of data from multiple diagnostics such as DXA and X-ray imaging [37], [39], [40], and the aggregation of risk factors [37]–[39].

In the present pilot study, we have included additionally collected subject data (7 new subjects) and have employed a neural network approach to process the previously obtained and new data. We hypothesize that the incorporation of a neural network classifier will significantly improve the predictive power of the presented system compared to the initial method based on a simple threshold binary classifier approach. The data collected from the device is an entire frequency spectrum of a complex scalar propagation coefficient through the wrist $(S_{21}\left ({f }\right))$. A neural network classifier sorts the spectra from different subjects as osteopenic or healthy. The network is trained using one subset of the collected data and validated with a separate subset. The neural network provides a binary predictor based on the spectrum it is given, as to whether the subject is healthy or osteopenic/osteoporotic.

## II. Materials and Methods

### A. Radiofrequency Measurement Device

**FIGURE 1.**
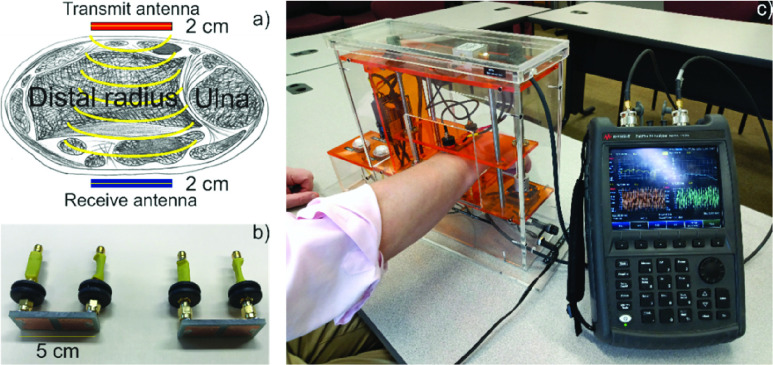
(a) Idealized diagram illustrating antenna placement on both sides of a human wrist. (b) Transmit and receive dual antiphase patch antennas with individual lumped-component matching networks. (c) Wrist tester device demonstration. Antenna length (along the wrist) is 5 cm; antenna width (across the wrist) is 1.8 cm. The antennas are fed in antiphase by a portable network analyzer and positioned by a lead screw for consistent 5 psi of pressure during measurement.

Fig. 1 (a) shows the arrangement of the two antennas transmitting through the wrist. These two antennas, Fig. 1 (b), are placed on the superior and inferior flat sides of the wrist adjacent to the position of the ulnar head. They are held in place with 1 kg of force during the recording of the measurements. The radiofrequency signal travels from the transmit antenna, through skin, bone, cartilage, and soft tissue to arrive at the receive antenna. Each of these layers provides some degree of attenuation and scattering; in the wrist, bone is significant compared to other body compartments. A network analyzer, Fig. 1 (c), measures the transmission coefficient $S_{21}\left ({f }\right)$ over the 300 kHz to 2 GHz range. This transmission coefficient is correlated to osteopenic and osteoporotic conditions. Details of the design of the system have been published previously in [32].

### B. Data Collection

After receiving Institutional Review Board (IRB) approval (IRB-19-0123) through Worcester Polytechnic Institute on Oct. 1, 2018, written informed consent was obtained from 80 subjects to participate in this pilot study (age range 23–94 years old, 60 female, 20 male). All measurements were further performed following the relevant IRB guidelines and regulations. 72 subjects were measured in a previous study [32], and 8 new subjects were added for this study. From 80 subjects, we selected 67 subjects suitable for a dichotomous diagnostic set:

#### 1) Group 1 (Osteopenic/Osteoporotic)

27 subjects (24 female, 3 male). Subjects were characterized by a T-score less than −1 taken within one year. Subjects with a T-score less than −2.4 were considered osteoporotic while other subjects were considered osteopenic. Subjects aged from 55 to 90 years with a mean of 77.5 and a standard age deviation of 10.1 years.

#### 2) Group 2 (Healthy)

40 subjects (26 female, 14 male). Subjects in this group did not necessarily have a known T-score, but instead were characterized by having none of the following risk factors: a history of bone fractures, medication for bone-related diseases, a family history of bone fractures, and/or osteoporosis. Subjects aged from 23 to 94 years with a mean of 60.2 and a standard age deviation of 16.6 years. It is noteworthy that these clinical risk factors can have a larger impact on fracture risk than one standard deviation decline in bone density [41], [42]. Therefore, we are comfortable considering them at low risk without explicit BMD information.

Location on the body of DXA tests and ongoing medications were not considered when assigning subjects to the groups.

**FIGURE 2.**
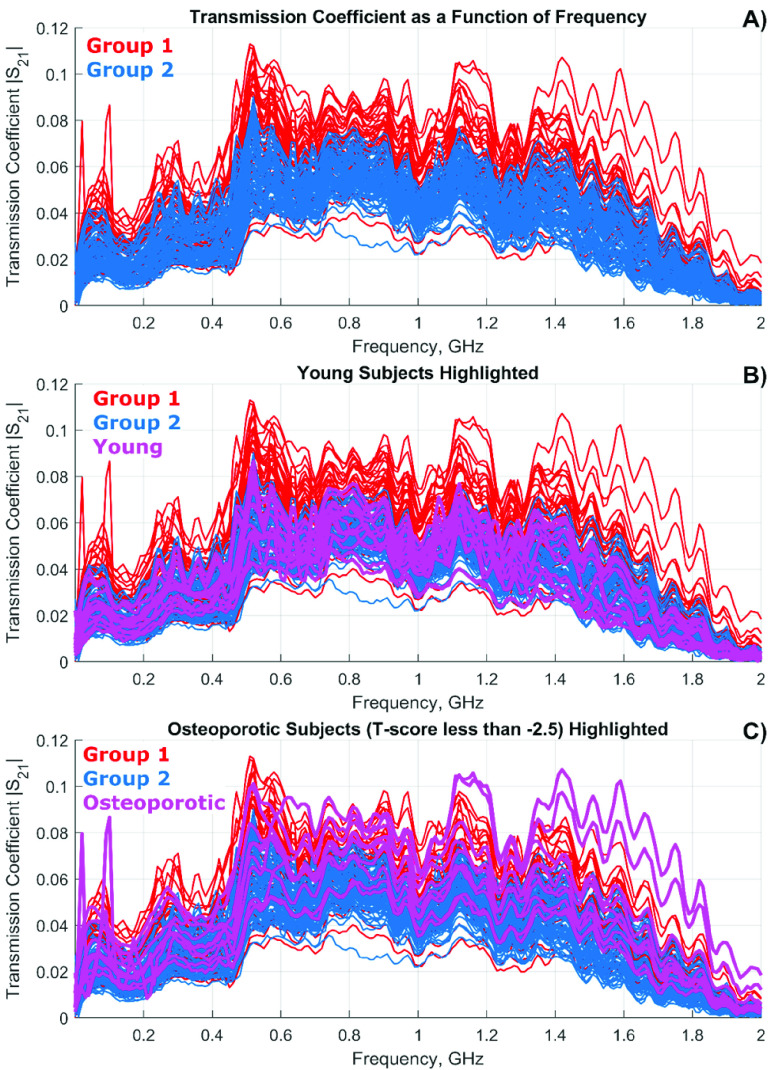
(a) Transmission coefficient ${\vert S}_{21}\left ({f }\right)\vert $ between the two antennas and through left and right wrists the frequency range 0–2 GHz for all subjects from Group 1 (osteopenic/osteoporotic) and Group 2 (healthy). The red color corresponds to Group 1 while the blue color corresponds to Group 2. 160 frequency curves (both arms for all 80 subjects) in total are shown in the figure. (b) The same as in a) but with the data for seven young adults highlighted in magenta. (c) The same as in a) but with the data for five osteoporotic subjects (T score below–2.5) highlighted in magenta.

Each subject’s data consists of their wrists’ circumferences in cm and four 201-point spectra: the transmission coefficient $\left ({S_{21} }\right)$ and the reflection coefficient $\left ({S_{11} }\right)$ for both left and right wrists. Fig. 2A shows the magnitude of the transmission coefficient, $\left |{ S_{21}\left ({f }\right) }\right |$ for 201 frequency sampling points between 300 kHz and 2.0 GHz. Group 1 is plotted in red, and Group 2 is plotted in blue. Fig. 2B has seven young subjects (age 44 and below) highlighted in magenta. Fig. 2C has five osteoporotic subjects with a DXA T-score below −2.4 highlighted in magenta.

The network analyzer recorded each spectrum as two components: magnitude and phase. For use with the neural network, the magnitude and phase were combined to give the complex number representation (real and imaginary component for each point) of each spectrum.

An additional set of data was created by normalizing the raw data described in the paragraphs above by risk factors of osteoporosis. The normalization factor was the subject’s age divided by their body mass index, as used in Eqn. 1 below to calculate the normalized $S_{21}$ spectrum.

\begin{equation*} S_{21N}=\frac {Age}{BMI}S_{21}\tag{1}\end{equation*}

### C. Neural Network Topology

**FIGURE 3.**
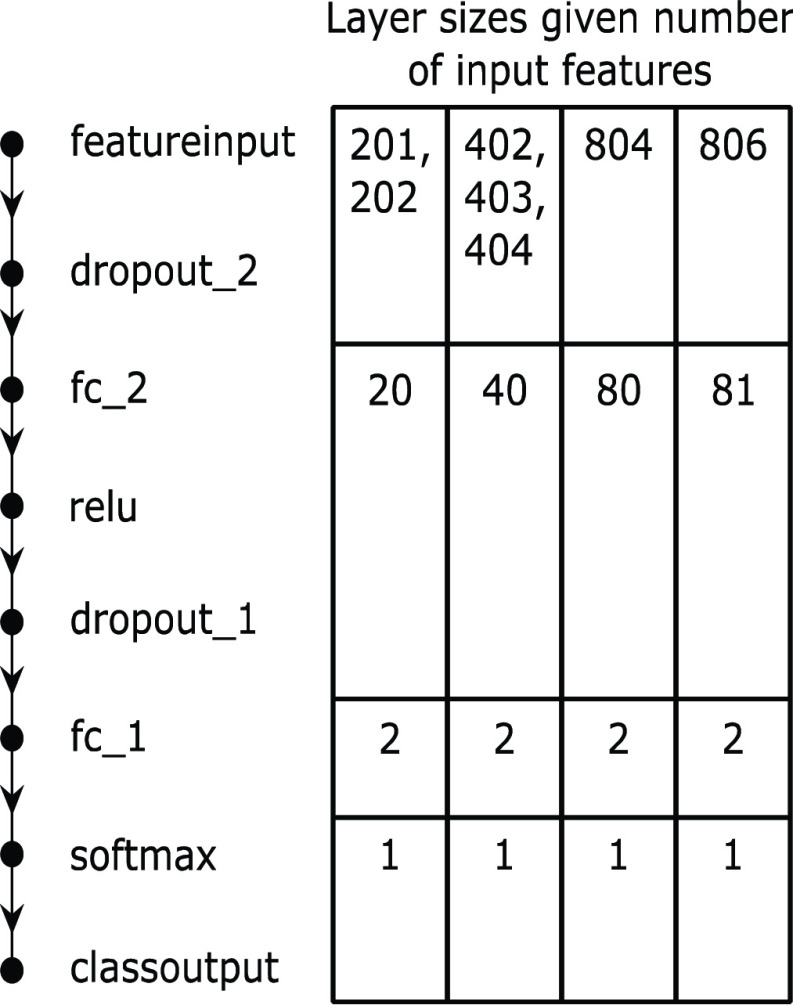
MLP classification neural network flow diagram featuring two fully connected layers.

The neural network used to generate the binary classifier was a multilayered network based on a Multi-Layer Perceptron (MLP) classifier implemented using the MATLAB Deep Learning Toolbox^®^ (MathWorks, Inc., Natick, MA, USA). Fig. 3 shows a flow diagram of this neural network. A featureinput layer read in the spectrum. The first 50% dropout layer, dropout_2, prevented overfitting of the first fully-connected layer (fc_2) by setting each feature to 0 with a 50% probability. fc_2 had unit learn rate factors for all weights and biases, its weights were L2 normalized, and its biases were not. Its weights were initialized using Glorot’s algorithm [43] and its biases were initialized to 0. reduced the number of features according to Eqn. 2, where $N_{in}$ is the number of input features (equal to the number of points in the spectrum for this layer) and $N_{out}$ is the number of neurons and output features from the layer.

\begin{equation*} N_{out}\mathrm {=nint}\left ({N_{in} \mathord {\left /{ {\vphantom {N_{in} {10}}} }\right. } {10} }\right)\mathrm {, 201\le }N_{in}\le 806\tag{2}\end{equation*}

A relu activation function separated the first and second layers and the second 50% dropout layer, dropout_1, prevented the second fully-connected layer from overfitting. This layer, fc_1, reduces the number of features from $N_{out}$ to 2. fc_1’s learn rate factors and L2 normalization settings were identical to fc_2’s. Those two features are compared using a softmax function to determine the predicted classification (Group 1 or Group 2) for the subject. All non-mentioned parameters were left at their default values.

The final size of the vector presented to the neural networks depended on how the arms of the subject were being combined. The longest was 806 features when the complex arm spectra (402 features each) and both wrist circumferences were concatenated. The shortest, at 201 points, was made from a single spectrum (magnitude or phase).

### D. Training, Validation, and Classification

Three methods of combining the spectra from the left and the right arms to generate a single diagnosis for the subject were attempted. First, the left and right arm spectra were averaged before being processed by the neural network. Second, the left and right arm spectra were concatenated to form a double-length spectrum. Third, each arm’s spectrum was presented separately to the neural network and the results were combined manually during postprocessing. Neural networks were trained for all combinations of data features using all three arm combination methods.

These neural networks were trained using the same parameters regardless of their number of input features. All networks were trained for 1000 epochs. To evaluate the overall usefulness of a specific configuration of input data, the neural networks were analyzed using a leave-one-out cross-validation scheme. Under this scheme, the subjects were randomly distributed between 7 subsets while maintaining roughly proportional numbers of Group 1 to Group 2 for either 9 or 10 subjects in each subset. The neural network would then be trained 7 times using each subset as the validation data once while all 6 other subsets were used as training data. Therefore, each input data configuration resulted in 7 trained neural networks of identical size each with a different validation data set. The mean of the resulting sensitivities, specificities, and accuracies from these 7 networks was used to characterize the performance of that input data configuration. Another series of tests using 10 subsets (6 or 7 subjects each) was attempted but did not yield results significantly different from the 7-subset tests.

The case wherein the two arms of each subject were presented separately to the neural network required an extra step after classification before the performance could be evaluated on a subject-by-subject basis. If both arms of a subject yielded the same classification, that classification was accepted. In the case of a conflict, the arms’ results were combined using each of four schemes: keep left, keep right, keep osteoporotic, keep healthy. In addition to the recombined results, the statistics were also computed as if each arm belonged to a separate subject.

## III. Results

Complex spectra produced the best results for raw data; magnitude spectra results are given for reference comparison. Combined magnitude and phase or phase-only number formats did not produce results worth including. Additionally, concatenating the arm spectra did not produce results worthy of inclusion. Using only the left or right arm of a subject produced results similar to those when the arms’ spectra were averaged.

### A. Non-Normalized Data

Table 1 shows the results of training the neural network using the output of the device directly. The only pre-processing involved in this data was done to put the complex data into the appropriate numerical representation – magnitude and/or phase versus complex number.

**TABLE 1:** Statistics for Neural Networks Trained From Raw (Non-Normalized) Transmission Data. The First 4 Rows Refer to Networks Trained Using 134 Subjects With 1 Arm Each While the Last 4 Rows Refer to Neural Networks Trained Using the Mean of the Left and Right Arm Spectra for Each of the 67 Subjects. Both Cases Were Investigated With and Without a Feature for the Subject’s Wrist Circumference Concatenated to the End of the Spectrum

Features	Subjects	Circumference	Format	Sensitivity	Specificity	Accuracy	Youden’s J
Left/right arm spectra as separate ‘subjects’	134	Concatenated	Complex	0.827	0.940	0.898	0.768
			Magnitude	0.780	0.917	0.867	0.696
		None	Complex	0.798	0.940	0.889	0.738
			Magnitude	0.780	0.929	0.875	0.708
Mean of arm spectra	67	Concatenated	Complex	0.690	0.905	0.824	0.595
			Magnitude	0.631	0.929	0.817	0.560
		None	Complex	0.690	0.905	0.824	0.595
			Magnitude	0.631	0.905	0.803	0.536

### B. Normalized Data

Table 2 shows the results of training the neural network using the data that was normalized according to Eqn. 1. First, the complex data was converted to the appropriate numerical representation – magnitude and/or phase versus complex number – then Eqn. 1 was applied to generate the values for the neural network.

**TABLE 2:** Statistics for Neural Networks Trained From Transmission Data That was Normalized According to Eqn. 1. The First 4 Rows Refer to Networks Trained Using 134 Subjects With 1 Arm Each While the Last 4 Rows Refer to Neural Networks Trained Using the Mean of the Left and Right Arm Spectra for Each Subject. Both Cases Were Investigated With and Without a Feature for the Subject’s Wrist Circumference Concatenated to the End of the Spectrum

Features	Subjects	Circumference	Format	Sensitivity	Specificity	Accuracy	Youden’s J
Left/right arm spectra as separate ‘subjects’	134	Concatenated	Complex	0.804	0.940	0.890	0.744
			Magnitude	0.804	0.917	0.875	0.720
		None	Complex	0.804	0.964	0.904	0.768
			Magnitude	0.780	0.917	0.867	0.696
Mean of arm spectra	67	Concatenated	Complex	0.810	0.929	0.884	0.738
			Magnitude	0.845	0.905	0.883	0.750
		None	Complex	0.762	0.952	0.883	0.714
			Magnitude	0.798	0.905	0.867	0.702

**FIGURE 4.**
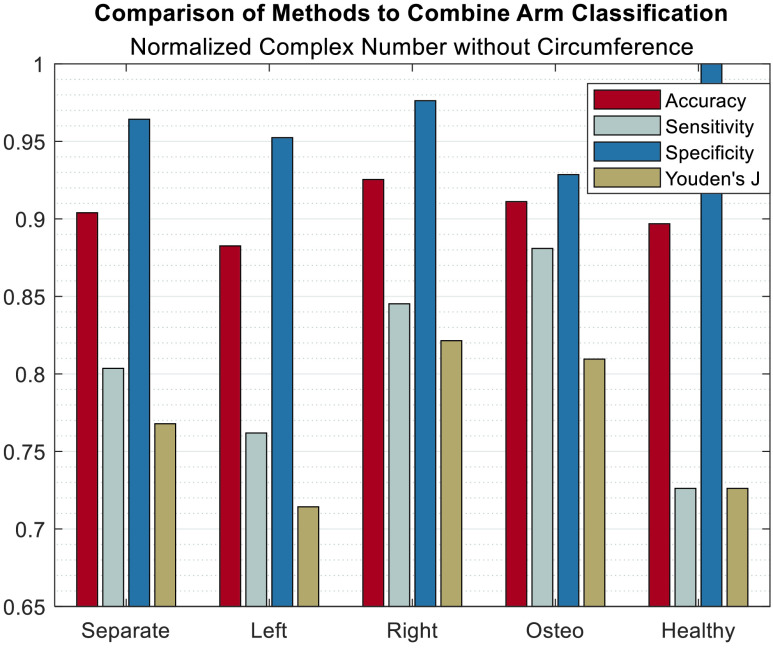
Comparison of methods for recombining 134 single-arm subjects to provide one classification per subject. ‘separate’ statistics are for the 167 single-arm ‘subjects’ while the other bar sets are sorted by what measurement was kept in case of a mismatch between the classification of the left and right arms’ data. ‘left’ and ‘right’ resolved mismatches by taking the result from the left or right arm, respectively. ‘osteo’ classified all mismatched subjects as group 1 and ‘healthy’ classified all mismatches as group 2.

Additionally, Fig. 4 shows a comparison of methods for recombining the classification results from the 134 single-armed ‘subjects’ from the highlighted row in Table 2 back into the 67 subjects that originated them. The same operation was also run using the data from Table 1 and similar performance was observed.

## IV. Discussion

In this pilot study, we have found that a neural network trained:

i with the complex frequency spectrum of radio wave propagation through the wrist and
ii with the wrist circumference

may serve as a promising predictor tool for detecting osteopenic/osteoporotic conditions on the wrist. Other recent studies have shown a strong correlation between bone density measurements in the arms, hip, and spine [44], [45]. Raw non-normalized data for the transmission coefficient through both wrists have been used as an input, without any normalization. This is in stark contrast to our initial study [32], where the processed data included the risk factors as well. In [32], a simple threshold binary classifier was used, which is essentially equivalent to checking the area under the entire frequency curve in Fig. 2 for every subject.

### A. Limitations of the Study

The limitations are as follows:

i All subjects in Group 1 had a DXA exam within 1 year of measurement, but the location of that exam and any following medications were not considered. Most Group 1 subjects were 70 years or older.
ii Subjects in Group 2 did not necessarily undergo a DXA measurement. Additionally, fracture data since our measurements were taken is not available for the majority of Group 2. Many of the subjects in Group 2 were young adults, age 18–25.
iii 13 subjects were not categorizable into Group 1 or Group 2 by all of their metrics simultaneously and were not considered for this study. For example, an elderly subject without a DXA exam in the past year.
iv The study considers the same single configuration of the measurement apparatus applied to two single body compartments (wrists).

Due to the lack of DXA measurements for many subjects in Group 2, we state only a partial similarity between our classification and DXA measurements. Incomplete fracture histories for Group 2 between the time of measurement and time of writing prevent any conclusions based on fracture history. Age differences between subjects can influence fat and muscle composition as well as bone composition, which could affect the classification. Different body compartments are composed of different amounts of fat, bone, and muscle so techniques that work well in one (for example the wrist, which is mostly bone) may not be directly applicable (as of today) to other more complex areas, such as the hip or spine. Because Group 2 had 10 more subjects than Group 1, sensitivity for a given trial is not as precise as specificity. We have used leave-one-out cross-validation to reduce the effects of this in our overall results.

### B. Fractures in Group 1

BMD data by DXA correlate with fracture risk but the correlation is not strong. To investigate this conclusion further, we collected data on fractures for subjects in Group 1 (osteopenic/osteoporotic). Except for one subject who deceased, two out of the 27 have experienced fractures over the last three years: one of the subjects – twice. All three cases were hip fractures. Four other elderly subjects in that group experienced falls without bone fractures over the last three years.

### C. Non-Normalized Data

The trained neural network provides sensitivity and specificity values of ~83% and 94%, respectively. The specificity compares favorably to the sensitivity and specificity provided by the inclusion of risk factors (both 87%), presented in a prior study [32]. The increase in the specificity obtained in the present study is a significant advantage due to the increased correctness when predicting the healthy condition, thereby improving utility for prescreening.

The improvement of the specificity is likely due to the use of the entire frequency information from Fig. 2. Neither the single integral over the entire frequency band nor a visual inspection of the multiple spectrum peaks can extract this additional information. On the other hand, the neural network classifier extracts additional useful features directly from the complex spectrum. These could be related to the relative positions and the relative peak values of several dominant spikes in Fig. 2.

The inclusion of the phase data by the neural network serves to increase its sensitivity compared to a network trained using only magnitude data. Further inclusion of wrist circumference increases both sensitivity and specificity by around 2% in most cases. This is likely due to wrist circumference being related to wrist fat content.

### D. Normalized Data

When the neural network is applied to the normalized dataset (which includes other risk factors as in [32], see Eqn. 1), a slight improvement is obtained. Normalizing the data provides a ~3% increase in overall accuracy and Youden’s index. This boost is only observed in data sets that do not include the wrist circumference; datasets including the wrist circumference exhibit a loss of performance. It appears, therefore, that inclusion of additional risk factors will be complementary to the ability of the transmission data to reliably differentiate between healthy and diseases patients. Networks trained from normalized data perform better *without* the inclusion of wrist circumference data, likely because the normalization and wrist circumference data perform the same role of predicting wrist fat content and/or bone size. No normalization techniques other than the one presented in Eqn. 1 were investigated.

## V. Conclusion

The present study reports the application of a neural network classifier to the processing of previously collected data on very-low-power radiofrequency propagation through the wrist to detect osteoporotic/osteopenic conditions. Our approach categorizes the data obtained for two dichotomic groups. Group 1 included 27 osteoporotic/osteopenic subjects with low BMD (DXA T score below - 1) measured within one year. Group 2 included 40 healthy and mostly young subjects without major clinical risk factors such as a (family) history of bone fracture.

We process the complex radiofrequency spectrum from 30 kHz to 2 GHz. Instead of averaging data for both wrists, we are processing them independently along with the wrist circumference and then combine the results, which greatly increases the sensitivity. Measurements along with data processing require less than 1 min. Neural network classifiers can identify and use characteristics of the data not readily apparent to the human eye to increase the specificity of predictions. The neural network classifier used in this study is a multilayer perceptron with two fully connected layers implemented with the help of MATLAB Deep Learning Toolbox^®^. It was trained using the leave-one-out approach as described in the Materials and Methods section.

For the two dichotomic groups, the neural network classifier of the radiofrequency spectrum reports a sensitivity of 83% and a specificity of 94%. These results are obtained without the inclusion of any additional clinical risk factors. Given that other recent studies have shown a strong correlation between bone density measurements in the arms, hip, and spine [44], [45], the radio transmission data may be usable on their own as a predictor of bone density. Our approach has the potential for screening patients at risk for fragility fractures in the office, given the ease of implementation, small device size, and low costs associated with both the technique and the equipment.

## Supplementary Material

10.21227/0ew3-pm58 APPLICATION OF A NEURAL NETWORK CLASSIFIER TO RADIOFREQUENCY-BASED OSTEOPENIA/OSTEOPOROSIS SCREENING There is an unmet need for quick, physically small, and cost-effective office-based techniques that can measure bone properties without the use of ionizing radiation. The present study reports application of a neural network classifier to the processing of previously collected data on very low power radiofrequency propagation through the wrist with the goal to detect osteoporotic/osteopenic conditions. Our approach categorizes the data obtained for two dichotomic groups. Group 1 included 27 osteoporotic/osteopenic subjects with low BMD (DXA T score below - 1) measured within one year. Group 2 included 40 healthy and mostly young subjects without major clinical risk factors such as (family) history of bone fracture. We process the complex radiofrequency spectrum from 30 kHz to 2 GHz. Instead of averaging data for both wrists, we are processing them independently along with the wrist circumference and then combine the results, which greatly increases the sensitivity. Measurements along with data processing require less than 1 min. For the two dichotomic groups identified above, the neural network classifier of the radiofrequency spectrum reports a sensitivity of 83% and a specificity of 94%. These results are obtained without inclusion of any additional clinical risk factors. They justify that the radio transmission data are usable on their own as a predictor of bone density. This approach has the potential for screening patients at risk for fragility fractures in the office, given the ease of implementation, small device size, and low costs associated with both the technique and the equipment. https://ieee-dataport.org/documents/application-neural-network-classifier-radiofrequency-based-osteopeniaosteoporosis
